# Supplementary material for: AI-driven approaches for dysgraphia diagnosis using online and offline handwriting data: A comprehensive scoping review
Source: PLoS One. 2025 Dec 31;20(12):e0328722. doi: 10.1371/journal.pone.0328722 (PMC12755808; doi:10.1371/journal.pone.0328722)
Supplement: S1 File — (DOCX) [file pone.0328722.s002.docx]

**Supplementary Data Explanation**

**Excel file**

This supplementary Excel file contains comprehensive data supporting the systematic review and meta-analysis described in the manuscript. The file is organized into nine sheets, each providing specific information related to the literature search, study selection, and data extraction processes employed in our study. Here is a detailed overview of each sheet:

**1. Sheet 'Paper': This sheet contains a consolidated list of all studies initially screened during the literature search. It includes important information such as study titles, publication years, date of extraction, area of study, and exclusion reasons. This comprehensive listing helps in understanding the scope of literature considered for the review. Additionally, Avisa Fallah and Yazdan Vakili reviewed each paper to ensure its inclusion in our study. The sheet also provides exclusion reasons and the date of extraction.**

**2. Sheet 'Included_Paper': This sheet details the studies included in the final analysis. It is evident that Yazdan Zandiye Vakili extracts some of the included paper’s data, and Avisa Fallah extracts some others according to the data extractor column. This differentiation aids in recognizing the contributions of individual team members to the data extraction process. Also, you can see the date of data extraction on each specific paper.**

**3. Sheet 'Keyword': It presents the finalized list of keywords used to search literature across various databases. This sheet is crucial for replicating the search methodology used in our study.**

**4. Sheet 'Source': Contains detailed information about the selected sources for the literature search, including direct links to the search queries and specific search strategies used for each source.**

**5. Sheets 'IEEE_Xplore', 'PubMed', 'Springer', 'Scopus', 'WOS' (World of Science): Each sheet provides specific details on the papers extracted from the respective databases. The sheet for each database outlines which papers were considered, and both data extractors do all of them.**

**Additional Explanations**:

- **Access Instructions**: This supplementary material is available directly via contacting the corresponding author.
- **Inclusion and Exclusion Reasons**: All the papers, as shown in the PRISMA figure, are categorized based on whether they are review papers, related to the topic, or ultimately included or excluded. Some of the excluded papers were not specifically about dysgraphia itself. Instead, they focused on related topics, such as analyses involving EEGs or brain images, rather than handwritten images. Others were excluded because they were review papers rather than empirical studies.
- **Quality assessment:** The quality of the included studies is assessed in the paper, and all the explanations can be found in section 3.4 (Results of Critical Appraisal within Sources of Evidence).
- **Data Protection and Privacy Compliance**: All data included in this file have been anonymized and prepared in compliance with applicable data protection and privacy regulations. No personal or sensitive information is disclosed.
- **Version and Date**: This document is the final version, created on 20/03/2024, and the final updates were applied on 27/05/2024. For any questions or corrections post-publication, please contact the corresponding author.
- **Use and Navigation Tips**: Users are encouraged to review the 'Sheet Guide' included within the file for tips on navigating and understanding the contents effectively. Each sheet is designed to be self-explanatory, with headings and notes guiding the data presented.

**Figures**

All the figures in the paper are included separately in the supplementary files. All eleven figures are either created by us or are accessed, and you can see the reference of that in the paper.
