# Supplementary material for: AI-driven approaches for dysgraphia diagnosis using online and offline handwriting data: A comprehensive scoping review
Source: PLoS One. 2025 Dec 31;20(12):e0328722. doi: 10.1371/journal.pone.0328722 (PMC12755808; doi:10.1371/journal.pone.0328722)
Supplement: Attachment — Submitted filename: RESPON~1.PDF [file pone.0328722.s005.PDF]

Note: To enhance clarity, we have highlighted the reviewers' comments and used **Arial font** for them, while our responses are provided in **Times New Roman**.

Third Revision

Reviewer #1: Most of the comments in the revised version have been properly addressed and no further comments are needed.

Reviewer #2: The authors have made commendable efforts to revise the manuscript in response to my previous comments, and several aspects of the paper have indeed improved. However, I believe further refinements are necessary. Some of the earlier comments appear to have been addressed only in a general or superficial manner, lacking the specificity and depth required to significantly enhance the quality. Therefore, additional revision is recommended. In this round of feedback, I have provided more detailed and structured suggestions to help strengthen the manuscript further. Additionally, I have included one new comment (Comment number 5).

1. Regarding your response to my second comment in the first revision:

My comment was : "While your scoping review provides insights into AI-based models for dysgraphia diagnosis, it currently lacks a discussion of existing review/survey papers on this topic. This omission limits the reader's ability to understand how your work builds upon or differs from previous efforts in the field. Include a section that identifies and briefly summarizes existing review papers related to AI-based dysgraphia diagnosis."

While I appreciate the addition of a discussion on previous review papers in Section 3.2, the response remains too general and does not fully address the concern. The discussion broadly mentions limitations of past reviews without explicitly citing and engaging with specific existing surveys on AI-based dysgraphia diagnosis. For example, one recent and comprehensive review (https://link.springer.com/article/10.1007/s10032-024-00464-z) has examined AI methodologies for dysgraphia diagnosis, and provided a structured analysis of automated systems, yet your manuscript does not acknowledge or differentiate itself from this prior work. Instead, it makes broad claims about the shortcomings of earlier reviews without demonstrating how your study adds new insights beyond what has already been explored. To strengthen the scholarly rigor of your manuscript, I recommend explicitly citing and summarizing existing review papers rather than referring to them in a generalized manner, clearly articulating how your review extends, updates, or differs from prior work, and avoiding generalized critiques of previous reviews unless supported by direct comparisons with specific papers. A more detailed engagement with prior surveys will provide a clearer justification for your review and ensure it is properly positioned within the existing body of literature.

Also i suggest changing the title of section 3.2.

Thank you for your insightful comment regarding the clarity of the literature gap our review aims to address. We have revised Section 3.2 to more explicitly highlight how our work fills key gaps left by prior literature. Specifically, unlike earlier reviews that tend to either focus broadly on handwriting difficulties or lean heavily toward clinical perspectives, our review is dedicated exclusively to dysgraphia and places emphasis on artificial intelligence-based diagnostic methods. We address the lack of comprehensive analysis across both traditional machine learning techniques (e.g., SVM, Random Forest) and emerging deep learning architectures (e.g., CNNs).

Additionally, we point out that many earlier reviews overlook modern AI approaches or fail to differentiate between dysgraphia and related learning disabilities. By contrast, our paper aims to provide a focused, up-to-date synthesis of AI-based dysgraphia research. To support this clarification, we have incorporated comparisons with existing reviews and added relevant references where appropriate. These revisions enhance the clarity of our paper's unique contribution to the field.

----

2. While the added discussion on evaluation metrics (Section 4.1.2) is a valuable improvement, the section would benefit from further expansion on the clinical and practical implications of metric choices. Specifically, it would be useful to discuss how different metrics align with real-world dysgraphia diagnosis scenarios, considering the trade-offs between false positives and false negatives. Additionally, while the discussion acknowledges metric heterogeneity, it would be strengthened by proposing potential future directions for standardization and best practices in evaluating AI models for dysgraphia.

For section 4.1.2, we have significantly expanded this section to incorporate the clinical and practical implications of evaluation metrics in the context of dysgraphia diagnosis. We now clearly explain how different metrics align with real-world use cases. For example, we emphasize that recall (sensitivity) is crucial in school-based screening tools, where the primary goal is to avoid missing at-risk children who may need early intervention. Conversely, precision becomes more critical in clinical settings, where minimizing false positives helps prevent unnecessary diagnoses and the stress or resource strain that may follow.

We also elaborated on the trade-offs between false negatives and false positives, outlining how they can directly affect intervention timing, educational support, and parental response. To address the challenge of metric heterogeneity across studies, we propose that future research adopt a core set of standardized evaluation metrics (e.g., recall, precision, F1-score, AUC), used consistently across tasks and datasets. We also recommend incorporating public benchmark datasets, k-fold cross-validation, and threshold tuning as standard best practices. Lastly, we underscore the value of interdisciplinary collaboration—including clinicians, educators, and AI researchers—in establishing evaluation standards that are both technically robust and practically meaningful. These additions strengthen the section's relevance and practical utility.

----

3. While the inclusion of a dedicated section (3.6) on feature analysis is a valuable addition, I believe there is still an opportunity to provide more depth and specificity. The categorization of features into temporal, spatial, pressure-based, and kinematic is a useful framework, but a more detailed discussion of how these features vary in the context of both online and offline handwriting could add significant value. Given the differences between these two modalities, it would be helpful to explore trends in feature selection across studies focusing on each type of handwriting separately. Additionally, discussing the relationship between feature selection and model performance in more detail—perhaps with specific examples from the reviewed studies—would help clarify how different features contribute to model efficacy.

Including relevant citations to support the claims made in this section would also strengthen the discussion, grounding the analysis in the broader body of literature. A brief overview of challenges related to feature standardization across studies would further enrich this section and highlight potential directions for future research. Overall, while the section is a good starting point, expanding on these aspects will provide readers

with a more comprehensive understanding of the role that feature selection plays in the development of AI-based dysgraphia diagnostic models.

For your constructive feedback on Section 3.6, we have substantially revised and expanded the section to address each of your suggestions in greater detail.

First, we retained the original categorization of features (temporal, spatial, pressure-based, and kinematic) and developed a more detailed comparison of how these feature types differ between online and offline handwriting modalities. For each feature category, we now provide modality-specific explanations that reflect differences in data acquisition, precision, and diagnostic utility. This includes the limitations of inferring temporal and kinematic features from offline data, as well as the enhanced granularity available from digital tablets in online modalities.

Second, we have improved subsection (3.6.2) discussing feature selection trends across studies, distinguishing between those focused on online handwriting, offline handwriting, and hybrid approaches. This trend analysis draws from specific studies reviewed in our work, with new in-text examples and citations (e.g., [21], [31], [34], [79]) that illustrate which features were selected and how they impacted model performance. For instance, one study achieved a 15% sensitivity improvement by incorporating kinematic features, while another reported high accuracy using spatial features alone in offline datasets. These examples help clarify the performance implications of different feature types, as you suggested.

Third, we have expanded Section 3.6.3 to more thoroughly discuss the relationship between feature selection and model efficacy. We now provide concrete performance outcomes from reviewed studies and explain how certain features align with clinically observable symptoms of dysgraphia (e.g., erratic pressure or prolonged stroke latency), contributing to both diagnostic performance and interpretability.

Fourth, as you recommended, we introduced a brief discussion of feature standardization challenges, including variation in tablet sampling rates, device pressure sensitivity, and inconsistent feature definitions across studies. This lack of standardization presents a barrier to reproducibility and comparability across models and was added to highlight future research needs.

Finally, we ensured that these revisions are supported by relevant citations to anchor our analysis in the broader literature. These updates strengthen Section 3.6 by providing the specificity, depth, and empirical grounding necessary to help readers better understand the pivotal role of feature selection in AI-based dysgraphia diagnosis.

4.The recommendations outlined in future works section provide a solid foundation for advancing dysgraphia detection models. The focus on combining individual fusion models with traditional machine learning approaches, addressing dataset limitations, and leveraging CNNs for improved performance are highly relevant and aligned with current research trends. However, a few aspects could benefit from further elaboration. For example, while the synergy between fusion models and traditional methods is well-highlighted, additional context or examples on how such integration could be practically implemented would strengthen this point. It would also be useful to address potential challenges, such as computational complexity or data requirements, associated with this integration. Regarding dataset limitations, the

mention of data augmentation methods is insightful, but a more detailed explanation of how these techniques can be specifically applied to handwriting data in the context of dysgraphia would be beneficial. Additionally, discussing how future studies could ensure that datasets are more diverse in terms of demographics, language, and cultural context would help provide a broader perspective.

Moreover, referencing studies that have successfully employed fusion models, advanced data augmentation, or CNNs in handwriting or related tasks could give a clearer picture of how these methods can be translated into dysgraphia detection. This would also demonstrate that the proposed future work is well-supported by the current body of research, giving it more credibility and a stronger basis for consideration.

We greatly appreciate your thoughtful feedback on the future works section and the encouragement to provide further elaboration. In response, we have revised the section to strengthen the depth and clarity of our recommendations, addressing all key points raised.

To elaborate on the integration of fusion models with traditional machine learning, we now describe how handcrafted or statistical features, extracted from traditional preprocessing pipelines (e.g., kinematic or pressure-based analysis), can be combined with deep feature representations and passed into conventional classifiers such as SVMs or Random Forests. This hybrid approach enables the integration of domain knowledge with learned representations, resulting in more robust and interpretable models. However, we also acknowledge potential challenges, such as increased computational complexity, limited scalability, and the difficulty of optimally weighting heterogeneous feature sources. These trade-offs are now explicitly discussed to give a more balanced and practical outlook.

Regarding dataset limitations and augmentation, we expanded the discussion to clarify how techniques such as shearing, shifting, mirroring, and flipping can simulate natural variations in handwriting, including stroke rhythm, pressure, and alignment—all of which are known indicators of dysgraphia. This specificity helps ground the relevance of data augmentation strategies to dysgraphia-related motor and spatial irregularities.

Additionally, we have addressed the need for greater dataset diversity by emphasizing the importance of including samples from diverse demographics, cultural contexts, and languages. We also expanded on dataset diversity via multi-center collaborations and non-Latin scripts (e.g., Arabic, Devanagari), ensuring inclusivity and reducing bias. This broader inclusion is crucial for enhancing generalizability and mitigating algorithmic bias.

To support these future directions, we have incorporated citations to studies that have successfully implemented fusion strategies, augmentation techniques, and CNN architectures in handwriting and related tasks. These references demonstrate the feasibility and prior success of the methods discussed, reinforcing the credibility of the proposed directions.

---

5. While Tables 6 to 11 present general advantages and disadvantages of the SVM, KNN, Random Forest algorithms etc..., they would benefit from a more focused discussion contextualized to dysgraphia diagnosis. For example, SVM's effectiveness with high-dimensional but small handwriting datasets has been reported in [Ref], while KNN's simplicity makes it a common baseline in early-stage studies despite its sensitivity to noise. Random Forest's ability to handle mixed feature types (e.g., spatial, temporal,

pressure-based) has proven useful in multimodal handwriting analysis [Ref]. Including such application-specific insights would enhance the relevance of these comparisons and help readers understand the trade-offs in algorithm selection for this domain.

Thank you for your valuable feedback. To fully address Comment 5, we have revised Tables 6–11 to include dysgraphia-specific insights in the advantages and disadvantages of each algorithm (SVM, KNN, Random Forest, K-means, AdaBoost, CNN), focusing on their application to handwriting data and dysgraphia diagnosis challenges. Specifically, we:

1. Updated Table Titles: Changed the titles of Tables 6–11 to explicitly reference dysgraphia diagnosis (e.g., Table 6: “Advantages and Disadvantages of SVM Algorithm in Dysgraphia Diagnosis”) to clarify the context.
2. Added Dysgraphia-Specific Insights: Incorporated application-specific advantages and disadvantages, such as SVM’s effectiveness with small, high-dimensional handwriting datasets, KNN’s role as a baseline despite noise sensitivity, Random Forest’s ability to handle multimodal features, K-means’ clustering of dysgraphic patterns, AdaBoost’s enhancement of weak classifiers, and CNN’s strength in complex image patterns despite dataset limitations.
3. Included Trade-off Discussion: Added a new paragraph at the end of Section 3.5 to discuss trade-offs in algorithm selection, highlighting how dataset characteristics (e.g., small sample sizes, multimodal features) and application constraints (e.g., computational resources) influence model choice for dysgraphia diagnosis.

These revisions ensure the tables and discussion are tailored to dysgraphia diagnosis, enhancing relevance and clarifying trade-offs, as requested.

Second Revision

2. We note that Figure 11 in your submission contain copyrighted images. All PLOS content is published under the Creative Commons Attribution License (CC BY 4.0), which means that the manuscript, images, and Supporting Information files will be freely available online, and any third party is permitted to access, download, copy, distribute, and use these materials in any way, even commercially, with proper attribution. For more information, see our copyright guidelines: http://journals.plos.org/plosone/s/licenses-and-copyright.

We require you to either (1) present written permission from the copyright holder to publish these figures specifically under the CC BY 4.0 license, or (2) remove the figures from your submission:

We would like to clarify that Figure 11, "Roadmap of Dysgraphia Detective Model Construction," is an original image created by us for Section 4.3 (Future Recommendations). This figure was developed specifically for this study and is not copied from any external source. Therefore, it fully complies with the CC BY 4.0 license and does not require additional permissions.

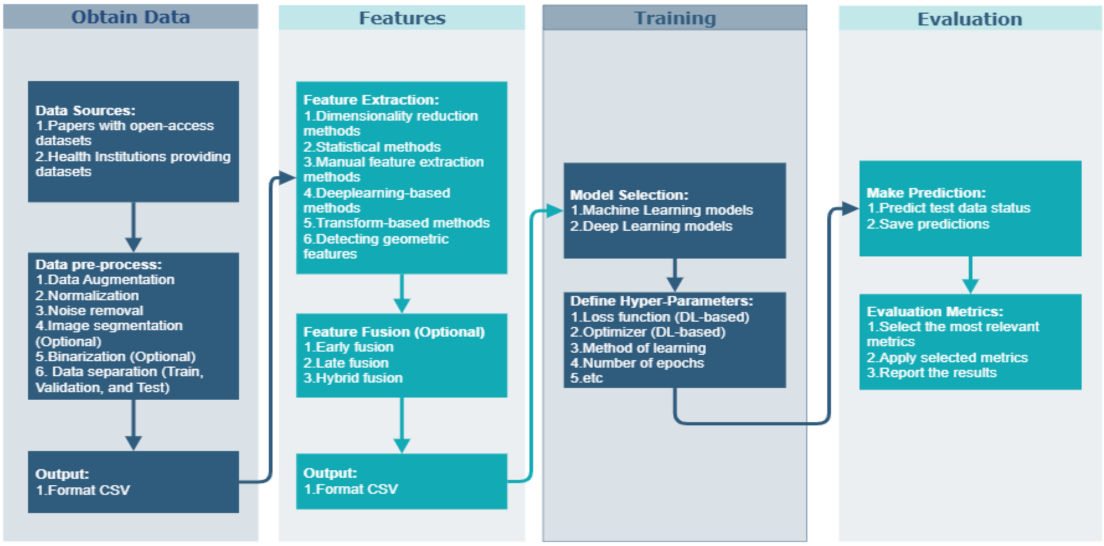

Fig 11 Roadmap of Dysgraphia Detective Model Construction

3. Please amend the title either on the online submission form or in your so that they are identical.

We have addressed this by ensuring that the title in the online submission form matches the title in the manuscript.

4. Please ensure that you refer to Tables 6-11 in your text as, if accepted, production will need this reference to link the reader to the Table

We have addressed this by ensuring that Tables 6–11 are properly referenced in the text to guide the reader accordingly.

5. Thank you for submitting your revised manuscript files to PLOS ONE. Before we can proceed, we kindly ask you to address the following concerns:

As required by our policy on Data Availability, please ensure your manuscript or supplementary information includes the following:

1) A numbered table of all 135 studies identified in the literature search, including those that were excluded from the analyses. For every excluded study, the table should list the reason(s) for exclusion.

2) Names of data extractors and date of data extraction.

This information can be included in the main text, supplementary information, or relevant data repository.

We have addressed this by including all required tables and explanations in our supplementary file titled *Supplementary-Data*. This file contains a numbered table of all 135 studies identified in the literature search, including reasons for exclusion where applicable. Additionally, it provides the names of data extractors and the dates of data extraction.

First Revision

1. Please ensure that your manuscript meets PLOS ONE's style requirements, including those for file naming. The PLOS ONE style templates can be found at

https://journals.plos.org/plosone/s/file?id=wjVg/PLOSOne_formatting_sample_main_body.pdf and

https://journals.plos.org/plosone/s/file?id=ba62/PLOSOne_formatting_sample_title_authors_affiliations.pdf

We have carefully reviewed the PLOS ONE formatting guidelines and ensured that our manuscript adheres to the required style, including file naming conventions.

---

2. When completing the data availability statement of the submission form, you indicated that you will make your data available on acceptance. We strongly recommend all authors decide on a data sharing plan before acceptance, as the process can be lengthy and hold up publication timelines. Please note that, though access restrictions are acceptable now, your entire data will need to be made freely accessible if your manuscript is accepted for publication. This policy applies to all data except where public deposition would breach compliance with the protocol approved by your research ethics board. If you are unable to adhere to our open data policy, please kindly revise your statement to explain your reasoning and we will seek the editor's input on an exemption. Please be assured that, once you have provided your new statement, the assessment of your exemption will not hold up the peer review process.

---

3. We note that Figure 11 in your submission contain copyrighted images. All PLOS content is published under the Creative Commons Attribution License (CC BY 4.0), which means that the manuscript, images, and Supporting Information files will be freely available online, and any third party is permitted to access, download, copy, distribute, and use these materials in any way, even commercially, with proper attribution. For more information, see our copyright guidelines: http://journals.plos.org/plosone/s/licenses-and-copyright.

We require you to either (1) present written permission from the copyright holder to publish these figures specifically under the CC BY 4.0 license, or (2) remove the figures from your submission:

1. You may seek permission from the original copyright holder of Figure 11 to publish the content specifically under the CC BY 4.0 license.

We recommend that you contact the original copyright holder with the Content Permission Form (http://journals.plos.org/plosone/s/file?id=7c09/content-permission-form.pdf) and the following text:

“I request permission for the open-access journal PLOS ONE to publish XXX under the Creative Commons Attribution License (CCAL) CC BY 4.0 (http://creativecommons.org/licenses/by/4.0). Please be aware that this license allows unrestricted use and distribution, even commercially, by third parties. Please reply and provide explicit written permission to publish XXX under a CC BY license and complete the attached form.”

Please upload the completed Content Permission Form or other proof of granted permissions as an "Other" file with your submission.

In the figure caption of the copyrighted figure, please include the following text: “Reprinted from [ref] under a CC BY license, with permission from [name of publisher], original copyright [original copyright year].”

2. If you are unable to obtain permission from the original copyright holder to publish these figures under the CC BY 4.0 license or if the copyright holder's requirements are incompatible with the CC BY 4.0 license, please either i) remove the figure or ii) supply a replacement figure that complies with the CC BY 4.0 license. Please check copyright information on all replacement figures and update the figure caption with source information. If applicable, please specify in the figure caption text when a figure is similar but not identical to the original image and is therefore for illustrative purposes only.

We have addressed this issue by removing and replacing the copyrighted image with an open-access image from this source. The new figure adheres to the CC BY 4.0 license requirements. We have also updated the figure caption accordingly.

Before:

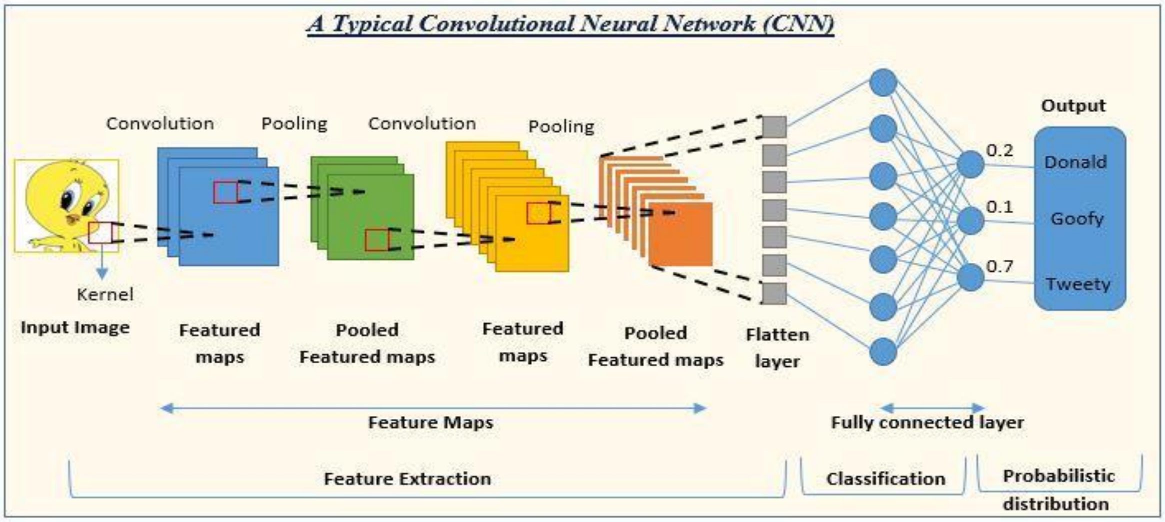

Fig 11 Sample of a typical CNN [67]

After:

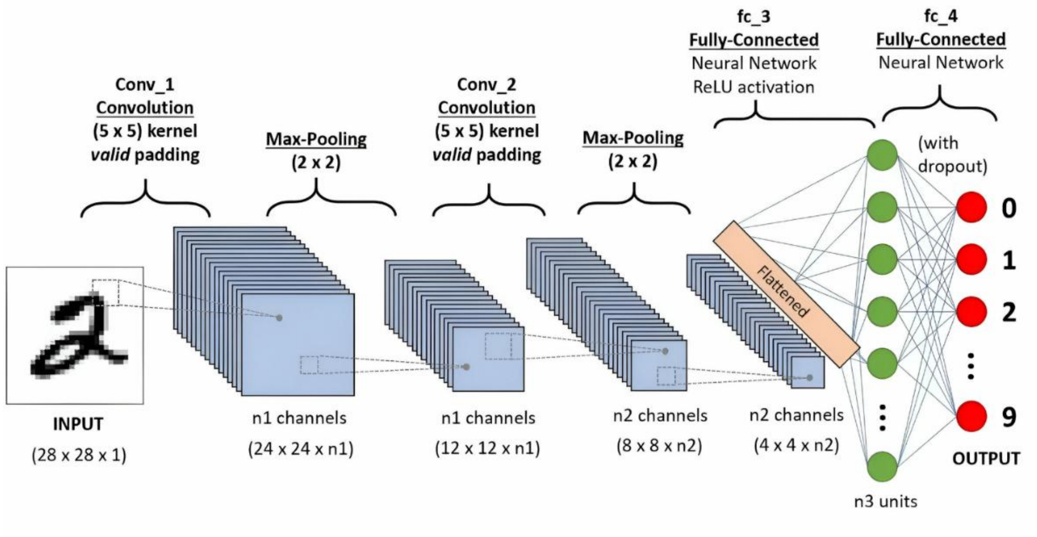

Fig 10 Sample of a typical CNN [67]

---

4. Please include captions for your Supporting Information files at the end of your manuscript, and update any in-text citations to match accordingly. Please see our Supporting Information guidelines for more information: http://journals.plos.org/plosone/s/supporting-information.

We have addressed this by including the captions for all Supporting Information files in a separate file named **"DysgraphiaPaper_Files"**, following the journal's guidelines.

---

5. As required by our policy on Data Availability, please ensure your manuscript or supplementary information includes the following:

A numbered table of all studies identified in the literature search, including those that were excluded from the analyses.

For every excluded study, the table should list the reason(s) for exclusion.

If any of the included studies are unpublished, include a link (URL) to the primary source or detailed information about how the content can be accessed.

A table of all data extracted from the primary research sources for the systematic review and/or meta-analysis. The table must include the following information for each study:

Name of data extractors and date of data extraction

Confirmation that the study was eligible to be included in the review.

All data extracted from each study for the reported systematic review and/or meta-analysis that would be needed to replicate your analyses.

If data or supporting information were obtained from another source (e.g. correspondence with the author of the original research article), please provide the source of data and dates on which the data/information were obtained by your research group.

If applicable for your analysis, a table showing the completed risk of bias and quality/certainty assessments for each study or outcome. Please ensure this is provided for each domain or parameter assessed. For example, if you used the Cochrane risk-of-bias tool for randomized trials, provide answers to each of the signalling questions for each study. If you used GRADE to assess certainty of evidence, provide judgements about each of the quality of evidence factor. This should be provided for each outcome.

An explanation of how missing data were handled.

We have addressed this by including all required tables and explanations in our supplementary file titled 'Supplementary-Data.' This file contains comprehensive data supporting our systematic review and meta-analysis, organized into nine sheets covering literature search, study selection, and data extraction. It includes a complete list of screened studies, reasons for inclusion/exclusion, keyword lists, search strategies, database-specific extractions, and a detailed breakdown of data

extraction responsibilities. Additionally, we provide explanations on quality assessment, data privacy compliance, and accessibility instructions. All figures from the manuscript are included separately in the supplementary files, with proper references provided.

For clarity, the detailed contents of 'Supplementary-Data' are also provided below:

Supplementary Data Explanation

Excel file

This supplementary Excel file contains comprehensive data supporting the systematic review and meta-analysis described in the manuscript. The file is organized into nine sheets, each providing specific information related to the literature search, study selection, and data extraction processes employed in our study. Here is a detailed overview of each sheet:

1. **Sheet 'Paper'**: This sheet contains a consolidated list of all studies that were initially screened during the literature search. It includes important information such as study IDs, authors, titles, publication years, and outcomes. This comprehensive listing helps in understanding the scope of literature considered for the review.
2. **Sheet 'Included_Paper'**: This sheet details the studies that were included in the final analysis. It features a color-coded system where entries marked in blue represent data extracted by Yazdan Zandiye Vakili, and entries in white are extracted by Avisa Fallah. This differentiation aids in recognizing the contributions of individual team members to the data extraction process.
3. **Sheet 'Keyword'**: It presents the finalized list of keywords that were used to perform the literature search across various databases. This sheet is crucial for replicating the search methodology used in our study.
4. **Sheet 'Source'**: Contains detailed information about the selected sources for the literature search, including direct links to the search queries applied and specific search strategies used for each source.
5. **Sheets 'IEEE_Xplore', 'PubMed', 'Springer', 'Scopus', 'WOS' (World of Science)**: Each of these sheets provides specific details on the papers extracted from the respective databases. The sheet for each database outlines which papers were considered and includes notes on the extractor, further illustrating the distribution of work among team members.

Additional Explanations:

- **Access Instructions**: This supplementary material is available directly via contacting the corresponding author.
- **Inclusion and Exclusion Reasons**: All the papers, as shown in the PRISMA figure, are categorized based on whether they are review papers, related to the topic, or ultimately included or excluded. Some of the excluded papers were not specifically about dysgraphia itself. Instead, they focused on related topics, such as analyses involving EEGs or brain images, rather than handwritten images. Others were excluded because they were review papers rather than empirical studies.

- **Quality assessment:** The quality of the included studies is assessed in the paper and all the explanations can be found in the section 3.4 (Results of Critical Appraisal within Sources of Evidence).
- **Data Protection and Privacy Compliance**: All data included in this file have been anonymized and prepared in compliance with applicable data protection and privacy regulations. No personal or sensitive information is disclosed.
- **Version and Date**: This document is the final version, created on 20/03/2024, and the final updates were applied on 27/05/2024. For any questions or corrections post-publication, please contact the corresponding author.
- **Use and Navigation Tips**: Users are encouraged to review the 'Sheet Guide' included within the file for tips on navigating and understanding the contents effectively. Each sheet is designed to be self-explanatory, with headings and notes providing guidance on the data presented.

Figures

All the figures in the paper, are included separately in the supplementary files. All of the eleven figures are either created by us or is open accessed and you can see the reference of that in the paper.

Reviewers' comments:

Reviewer's Responses to Questions

Comments to the Author

4. Is the manuscript presented in an intelligible fashion and written in standard English?

PLOS ONE does not copyedit accepted manuscripts, so the language in submitted articles must be clear, correct, and unambiguous. Any typographical or grammatical errors should be corrected at revision, so please note any specific errors here.

Reviewer #1: No

Reviewer #2: Yes

We have revised the manuscript to improve clarity, correctness, and readability. This includes refining the text, enhancing image explanations, and correcting grammatical and scientific errors. The manuscript is now presented in a clear, correct, and unambiguous manner.

---

5. Review Comments to the Author

Please use the space provided to explain your answers to the questions above. You may also include additional comments for the author, including concerns about dual publication, research ethics, or publication ethics. (Please upload your review as an attachment if it exceeds 20,000 characters)

Reviewer #1: The Title: AI-Based Models for Dysgraphia Diagnosis on Handwritten Images: A Comprehensive Scoping Review.

This paper provides a scoping review to explore available studies employing AI-based models for dysgraphia diagnosis and identify the highest performance models and their challenges to suggest future improvements. The work is good and interesting in its field. However, there are some points need to be considered which are as follows:

1- The authors stated that a scoping review is conducted instead of a systematic review with three mentioned objectives. However, scoping review needs to identify knowledge gaps, explain concepts and investigate research conduct. Are these concepts addressed in this manuscript? The authors should elaborate further to show what has been added to the academic field and what has been discovered and proven.

&

3- Some research questions need to be added to the manuscript. This type of review is broad because its purpose is to identify the scope of the literature on the presented topic, so, the research questions that this review can answer should be broad also.

We have made several important revisions to ensure the manuscript addresses these concerns effectively:

Clarification on Scoping Review Objectives:

We have added three new **objectives** (4, 5, and 6) to the introduction to further elaborate on the contributions of this review to the academic field and highlight the gaps discovered:

Objective 4: Examine the integration of machine learning techniques with traditional diagnostic methods.

This objective investigates how machine learning techniques are integrated with traditional diagnostic methods for dysgraphia, identifying the benefits and drawbacks of such integrations. It includes an analysis of hybrid models that combine AI with manual assessments or other diagnostic tools.

Objective 5: Analyze the training datasets used for developing AI models in dysgraphia diagnosis.

This objective analyzes the characteristics, diversity, and adequacy of the training datasets used in AI model development for dysgraphia diagnosis. It addresses the quality of data and its impact on model performance and generalizability.

Objective 6: Review technological advancements and their implications for future AI models in dysgraphia diagnosis.

This objective reviews recent technological advancements in AI and machine learning, including algorithm advancements, computational hardware, and data collection methods, and discusses their potential impacts on future models for dysgraphia diagnosis.

Before:

Objectives:
1- Identify available studies that used AI-based models for dysgraphia diagnosis, explore the current dominant use of AI in expediting dysgraphia detection.
2- Explore the challenges of building computational AI-based models under current conditions and the limitations of each study.
3- Determine the best models with high accuracy and performance in dysgraphia diagnosis and examine the effectiveness of these models in dysgraphia detection and possible future improvements in these models.

After:

Objectives:
1- Identify available studies that used AI-based models for dysgraphia diagnosis and explore the current dominant use of AI in expediting dysgraphia detection.
2- Explore the challenges of building computational AI-based models under current conditions and the limitations of each study.
3- Determine the best models with high accuracy and performance in dysgraphia diagnosis, examine their effectiveness in dysgraphia detection, and consider possible future improvements in these models.
4- Examine the integration of machine learning techniques with traditional diagnostic methods.
5- Analyze the training datasets used for developing AI models in dysgraphia diagnosis.
6- Review technological advancements and their implications for future AI models in dysgraphia diagnosis.

Enhanced Conclusions:

To align with the added objectives, we have expanded the **4.2. Conclusions section**. The following key points have been incorporated:

Integration with Traditional Methods: A detailed discussion on how the merging of machine learning with traditional diagnostic approaches can improve accuracy and reliability.

Training Dataset Analysis: A comprehensive analysis of the impact of dataset quality and size on the performance of AI models for dysgraphia diagnosis.

Technological Advancements: An overview of recent technological advancements and their potential for enhancing AI models in dysgraphia diagnosis.

Before:

This scoping review offers a thorough examination of the current landscape of AI-based models for dysgraphia detection through handwritten image analysis. The results indicate that AI models, particularly advanced machine learning models like CNNs and SVMs, can significantly enhance the diagnostic process for dysgraphia, offering more

objective, faster, and accurate assessments compared to traditional methods. This is crucial for mitigating the long-term impacts of dysgraphia on educational and social outcomes. This aligns well with the review objectives of identifying effective AI methodologies and understanding their application in real-world settings.

After:

This scoping review thoroughly examines the current landscape of AI-based models for dysgraphia detection through handwritten image analysis. The results indicate that AI models, particularly advanced machine learning models like CNNs and SVMs, can significantly enhance the diagnostic process for dysgraphia, offering more objective, faster, and more accurate assessments than traditional methods. This is crucial for mitigating the long-term impacts of dysgraphia on educational and social outcomes. This aligns well with the review objectives of identifying effective AI methodologies and understanding their application in real-world settings. Moreover, integrating machine learning techniques with traditional diagnostic methods has enhanced diagnostic accuracy and reliability synergistically. By incorporating machine learning outputs into conventional assessment frameworks, clinicians can better understand dysgraphia characteristics. The analysis of training datasets used in developing AI models revealed a significant variance in dataset quality and size, directly affecting model performance. Our review has identified a pressing need for standardized, large-scale datasets that more accurately reflect the diverse nature of handwriting styles across different demographics. Furthermore, ongoing technological advancements, such as the development of more sophisticated neural network architectures and enhanced computational power, promise to revolutionize the field of dysgraphia diagnosis further. These advancements will likely enable the creation of even more powerful and precise diagnostic tools in the near future.

Future Recommendations:

In response to the integration of machine learning with traditional methods (Objective 4), we have added the following explanation to the **4.3. Future Recommendations** section:

In addition to developing new models, there is a critical need to enhance the robustness of machine learning integration with traditional diagnostic tools. This can be achieved by refining the algorithms that process the input from these tools, improving the synergy between AI models and existing diagnostic methods.

These revisions ensure that the manuscript not only highlights the gaps in the existing literature but also outlines the potential opportunities for advancing AI in dysgraphia diagnosis, thus addressing the purpose of a scoping review. Furthermore, the addition of broader research questions is reflected in the new objectives, allowing the review to address various dimensions of the field.

2- It stated that “All original research articles published until April 2024 …….. were included.”. Is there only one research paper in 2024 submitted in this manuscript? Please review this issue.

In response to the observation regarding the sharp decrease in publications by 2024, the additional text in the revised manuscript addresses the likely bottleneck in advancing AI research in dysgraphia diagnosis: the scarcity of appropriate datasets. This limitation is critical as it directly impacts the development and validation of AI models. The decline to just one paper published in 2024 thus far underscores the urgent need for more robust and accessible datasets. This explanation

aims to clarify the observed publication trend and emphasize the importance of resolving data availability issues to continue progressing in this field.

We have added the following text to the **3.2. General Characteristics of the Included Papers** section:

"This global and varied contribution underscores the universal relevance and multidisciplinary nature of AI applications in dysgraphia diagnosis based on handwritten images, reflecting a widespread recognition of its potential impact across different educational and healthcare settings. Despite the broad interest, the recent slowdown in publications could signal a bottleneck in data availability, emphasizing the need for enhanced data collection and sharing efforts to sustain momentum in this crucial area of research."

4- Some references should be formatted according to the author's guidelines as there are many references that are not written correctly.

We have thoroughly reviewed and revised the references to ensure they comply with the journal's formatting guidelines. All references have been adjusted to meet the specified style, and any inconsistencies have been corrected.

5- There is no need to add [30] in the title of Figure 2 because the information provided is the author's own. Instead, [30] can be cited in the text of Section 3.1.

we have revised the caption of Figure 2 to remove the reference to [30] and to better reflect the content. The updated caption is now as follows:

Before:
Fig 2 The PRISMA diagram of study selection [30]

After:
Fig 2 Overview of Study Selection Using the PRISMA Framework

We have also ensured that reference [30] is properly cited within the text of Section 3.1 by adding the following sentence:

The screening process and data selection, as outlined in Fig 2, were based on PRISMA guidelines [30].

6- Figure 3 with the captions of its sub-figures needs more organization. In fact, the titles of most of the tables and figures needs to be reworded to make it more clear to the reader.

We have made the following revisions to improve readability and clarity.

For **Figure 3**, the caption has been reworded as follows:

Before:
Fig 3 General characteristic of included studies. A number of journal & conference articles; B Number of articles per year; C Number of articles per country; D number of public & private access studies

After:
Fig 3 General characteristics of the included studies. (A) Distribution of journal articles versus conference proceedings. (B) The number of publications by year shows the publication trends from 2015 to 2024. (C) Geographic distribution of studies by country. (D) Proportion of public versus private access to studies.

Additionally, we have applied similar revisions to other figures and tables in the manuscript (as indicated in our 'Revised Manuscript with Track Changes' file with comments) to ensure that their captions are clear, well-structured, and informative for the readers.

7- Ratios listed in figures that containing a circle should be as a percentage like the one in Figure 4.

We have revised the relevant figures to present the ratios as percentages, as shown in Figure 4. This adjustment ensures consistency and clarity across all figures containing ratios in circular representations.

Before:

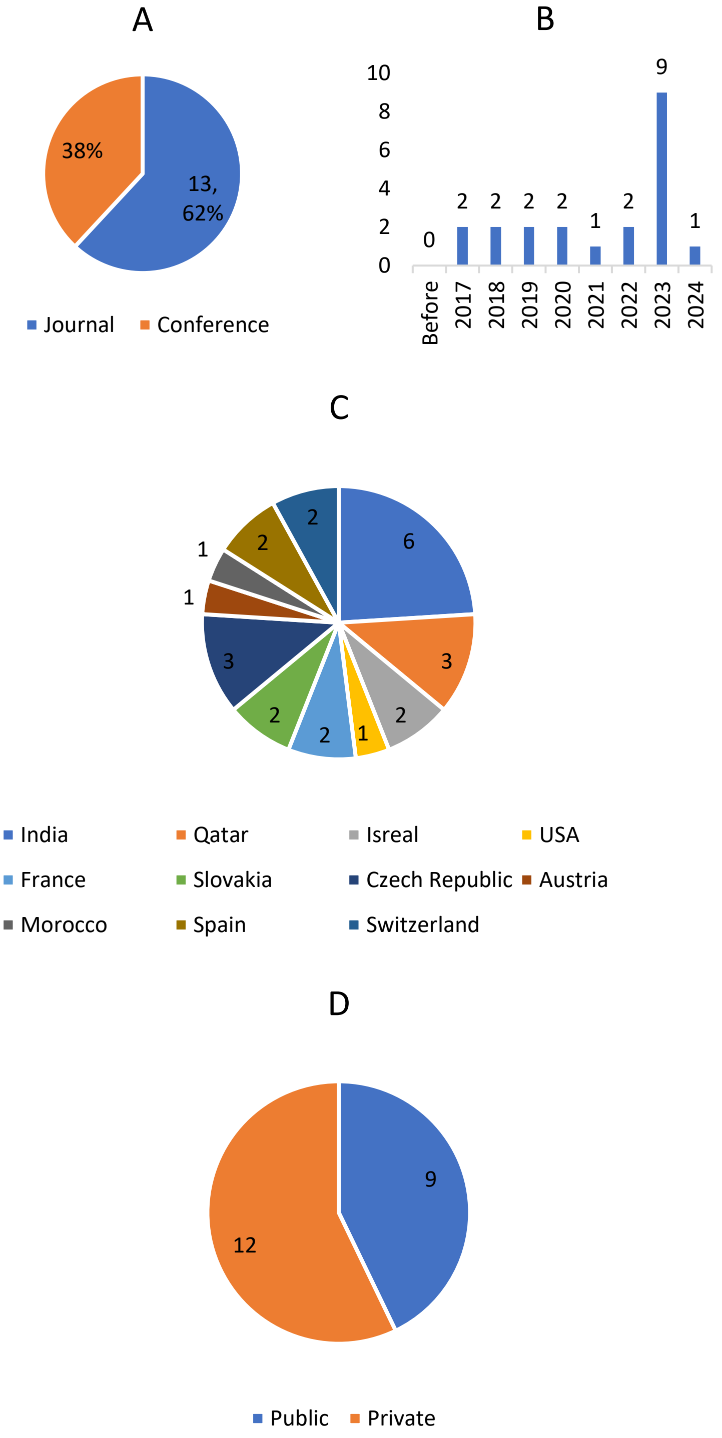

Fig 3 General characteristics of included studies. **A** number of journal & conference articles; **B** Number of articles per year; **C** Number of articles per country; **D** number of public & private access studies

After:

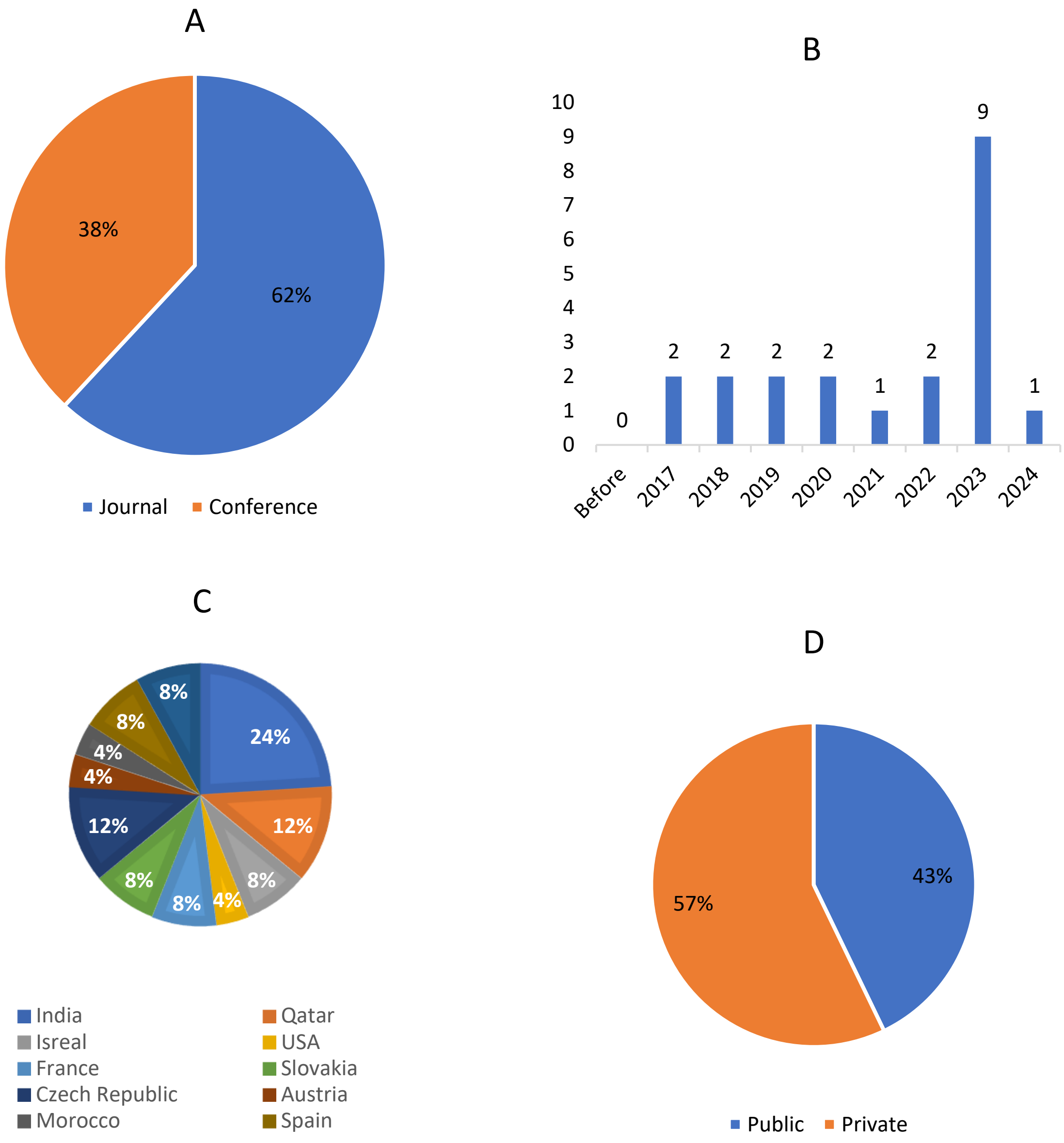

Fig 3 General characteristics of the included studies. **(A)** Distribution of journal articles versus conference proceedings. **(B)** The number of publications by year shows the publication trends from 2015 to 2024. **(C)** Geographic distribution of studies by country. **(D)** Proportion of public versus private access to studies.

Before:

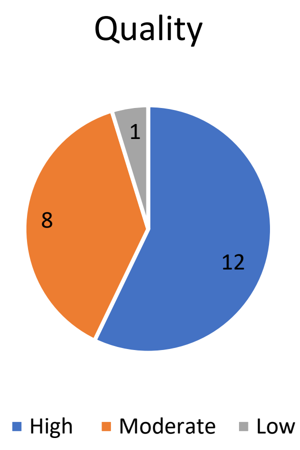

Fig 4 Included Papers Quality

After:

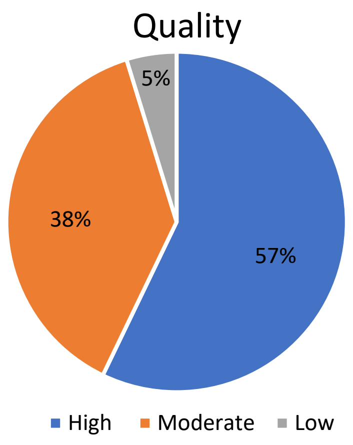

Fig 4 Quality Assessment Scores of Included Studies

8- There are many grammatical errors that should be checked and corrected in the entire manuscript.

We have thoroughly reviewed and corrected all grammatical issues throughout the manuscript. These revisions can be clearly seen in the 'Revised Manuscript with Track Changes' file, where all corrections have been indicated.

9- Figure 5 can be presented in another representation and the resolution of Figure 6 and Figure 7 should be increased.

In response to the comment about Figure 5, we have removed it from the manuscript and instead incorporated its content into Section 3.5, where we provide a detailed comparison of the most commonly used predictive models for dysgraphia detection.

Before:

3.5. Comparison of mostly used Predictive Models

This section will discuss the most widely applied models featured in the included papers (Fig 5). We aim to provide a comprehensive analysis by considering both their strengths and weaknesses. This balanced examination will help us understand the effectiveness and limitations of each model, thereby guiding the selection of the most appropriate techniques for our research objectives.

$$Predictive\ Models\ of\ Dysgraphia\ Detection \begin{cases} SVM \\ KNN \\ RF \\ K-Means \\ AdaBoost \\ CNN \end{cases}$$

Fig 5 The overview of existing models in Dysgraphia detection

After:

3.5. Comparison of mostly used Predictive Models

This section will discuss the most widely applied predictive models of dysgraphia detection featured in the included papers: SVM, KNN, RF, K-Means, AdaBoost, and CNN. We aim to provide a comprehensive analysis by considering both their strengths and weaknesses. This balanced examination will help us understand each model's effectiveness and limitations, thereby guiding the selection of the most appropriate techniques for our research objectives.

Regarding the resolution of Figures 6 and 7, we have replaced them with high-quality open-source images to ensure they meet publication standards.

Before:

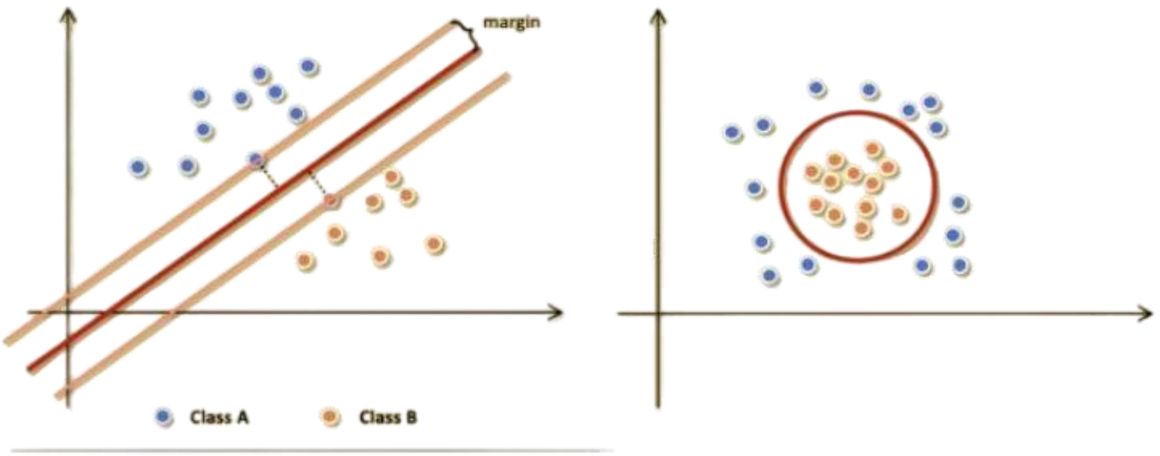

Fig 6 The example of linearly separable samples [53]

After:

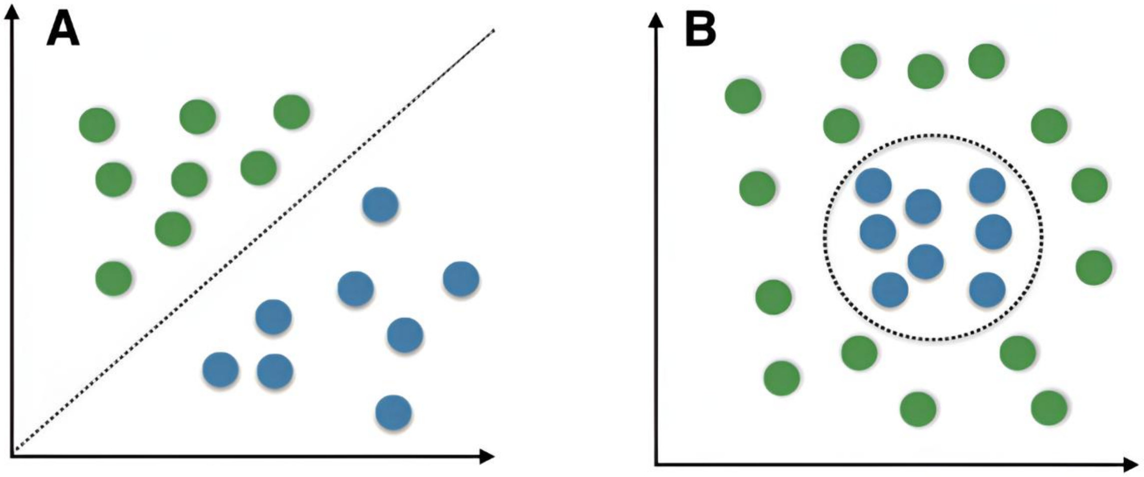

Fig 5 Examples of Linearly Separable Data Distributions [53]

Before:

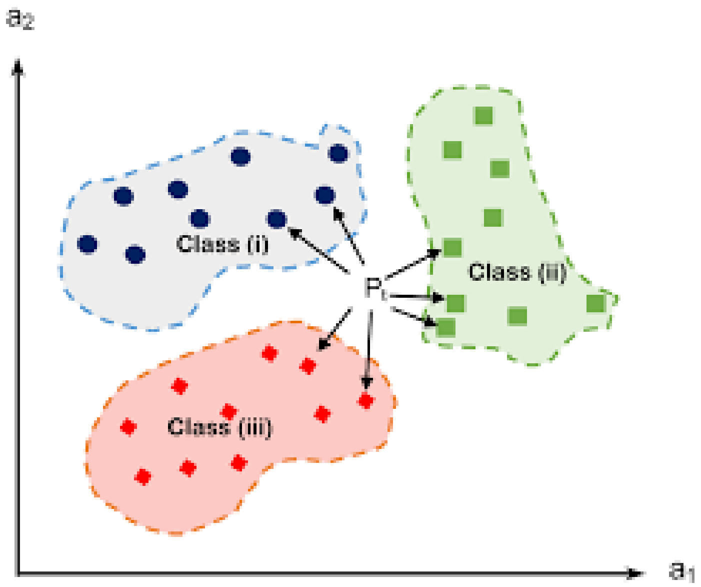

Fig 7 KNN algorithm overview [55, 56]

After:

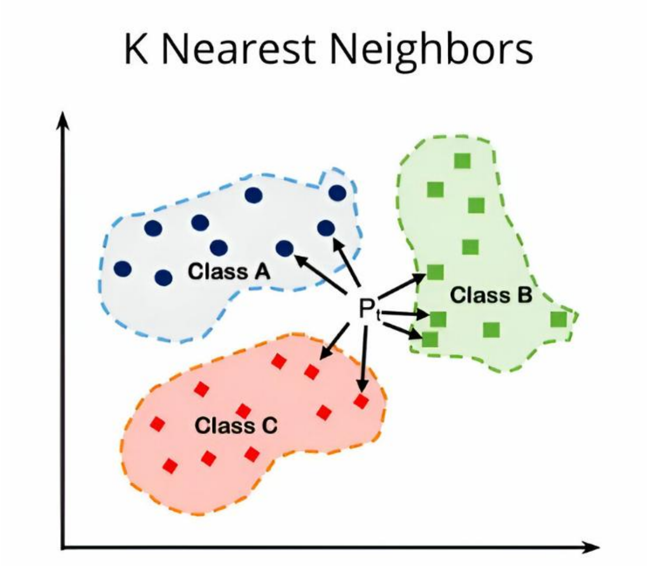

Fig 6 KNN algorithm overview [55, 56]

10- The section of the refences should have a title.

we have addressed it by adding a title to the section as follows:

Before: References

After: 5. References

Reviewer #2: This paper presents a comprehensive scoping review about AI based approaches in literature proposed for Dysgraphia diagnosis from handwritten data. The paper needs major improvements before publishing. Here are my recommendations:

1. The current title, "AI-Based Models for Dysgraphia Diagnosis on Handwritten Images: A Comprehensive Scoping Review," may not accurately reflect the full scope of the paper. While the review covers both offline (image-based) and online handwriting data methods, the title only mentions "handwritten images," which typically refers to offline data. Consider revising the title to encompass both types of data.

For example: "AI-Based Models for Dysgraphia Diagnosis Using Online and Offline Handwriting Analysis: A Comprehensive Scoping Review"

We have revised it to better reflect the full scope of the paper, which includes both offline (image-based) and online handwriting data methods. The title has been changed from:

"AI-Based Models for Dysgraphia Diagnosis on Handwritten Images: A Comprehensive Scoping Review"

to:

"AI-Driven Approaches for Dysgraphia Diagnosis Using Online and Offline Handwriting Data: A Comprehensive Scoping Review"

---

2. While your scoping review provides insights into AI-based models for dysgraphia diagnosis, it currently lacks a discussion of existing review/survey papers on this topic. This omission limits the reader's ability to understand how your work builds upon or differs from previous efforts in the field. Include a section that identifies and briefly summarizes existing review papers related to AI-based dysgraphia diagnosis.

In response to your valuable feedback, we have incorporated a detailed paragraph within section 3.2, 'General Characteristics of the Included Papers,' to discuss and compare previous review papers on AI-based models for dysgraphia diagnosis. This paragraph highlights the limitations of these earlier works, particularly their lack of a focused analysis on AI models, insufficient data on the datasets used, and the broad scope that often extends beyond dysgraphia. Our manuscript now provides a critical evaluation of how our review builds upon and differs from these prior efforts, enriching the overall context and emphasizing the novelty and specificity of our approach in the field.

Before:

3.2. General Characteristics of the Included Papers

The publication dates span nearly a decade, from 2015 to the present (Fig 3). Of the 21 selected papers, 8 (n = 8, 38%) are conference papers, and the remaining 13 (n = 13, 62%) are journal articles. The papers originate from various countries, including India, Qatar, Spain, Switzerland, Slovakia, the Czech Republic, Israel, France, the USA, Malaysia, Morocco, and Austria. Additionally, some papers are collaborative efforts involving researchers from multiple nationalities. In addition, only nine of the included studies are open-access (n=9, 42%), and the 12 other papers are private (n=12, 58%).

After:

3.2. General Characteristics of the Included Papers

The publication dates span nearly a decade, from 2015 to the present (Fig 3). Of the 21 selected papers, 8 (n = 8, 38%) [34,35,38,42-44,47] are conference papers, and the remaining 13 (n = 13, 62%) [20,31-33,36,39,40,41,45,46,48-50] are journal articles. The papers originate from various countries, including India, Qatar, Spain, Switzerland, Slovakia, the Czech Republic, Israel, France, the USA, Malaysia, Morocco, and Austria. Additionally, some papers are collaborative efforts involving researchers from multiple nationalities. In addition, only nine of the included studies are open-access (n = 9, 42%) [20,31-33,40,41,45,49], and the 12 other papers are private (n = 12, 58%) [34-39,42-44,46-48].

While our review provides detailed insights into the current AI methodologies for diagnosing dysgraphia, it is essential to contextualize this within the broader academic dialogue. Previous reviews and surveys have laid foundational knowledge in this area; however, they often fall short of providing a complete analysis focused exclusively on AI models. Many omit crucial details about the datasets used, and most importantly, they are not entirely dedicated to dysgraphia, often incorporating it as part of broader studies on learning

disabilities. Furthermore, the majority of these reviews are dated, with recent advancements in AI technologies scarcely reflected. Our work aims to fill these gaps, offering a dedicated and updated examination of AI applications in dysgraphia diagnosis, distinct from earlier efforts which have not comprehensively addressed these specific angles.

This global and varied contribution underscores the universal relevance and multidisciplinary nature of AI applications in dysgraphia diagnosis based on handwritten images, reflecting a widespread recognition of its potential impact across different educational and healthcare settings. Despite the broad interest, the recent slowdown in publications could signal a bottleneck in data availability, emphasizing the need for enhanced data collection and sharing efforts to sustain momentum in this crucial area of research.

----

3. There are several instances in the paper where specific studies or data points are mentioned without proper citation. For example:

"Two of the papers utilized multiple datasets (n = 2, 9%), while only three papers used raw handwritten images (n = 3, 14%)"

This statement provides specific numbers and percentages but fails to cite the actual papers being referenced. This oversight occurs in other parts of the manuscript as well. Carefully review the entire manuscript to identify and address all similar instances where specific studies or data points are mentioned without proper citation.

We have carefully reviewed the manuscript and addressed all instances where specific studies or data points were mentioned without proper citation. The relevant studies have now been properly cited in those sections to ensure that all data points are appropriately referenced:

Before:

The publication dates span nearly a decade, from 2015 to the present (Fig 3). Of the 21 selected papers, 8 (n = 8, 38%) are conference papers, and the remaining 13 (n = 13, 62%) are journal articles. The papers originate from a diverse range of countries, including India, Qatar, Spain, Switzerland, Slovakia, the Czech Republic, Israel, France, the USA, Malaysia, Morocco, and Austria. Additionally, some papers are collaborative efforts involving researchers from multiple nationalities. In addition, only nine of the included studies are open-access (n=9, 42%) and the 12 other papers are private (n=12, 58%).

After:

The publication dates span nearly a decade, from 2015 to the present (Fig 3). Of the 21 selected papers, 8 (n = 8, 38%) [34,35,38,42-44,47] are conference papers, and the remaining 13 (n = 13, 62%) [20,31-33,36,39,40,41,45,46,48-50] are journal articles. The papers originate from various countries, including India, Qatar, Spain, Switzerland, Slovakia, the Czech Republic, Israel, France, the USA, Malaysia, Morocco, and Austria. Additionally, some papers are collaborative efforts involving researchers from multiple nationalities. In addition, only nine of the included studies are open-access (n = 9, 42%) [20,31-33,40,41,45,49] , and the 12 other papers are private (n = 12, 58%) [34-39,42-44,46-48].

----

4. What is the need of figure 5. If you want to include it, then please redraw and represent it in a better way.

We have removed it from the manuscript and instead incorporated its content into Section 3.5, where we provide a detailed comparison of the most commonly used predictive models for dysgraphia detection.

Before:

3.5. Comparison of mostly used Predictive Models

This section will discuss the most widely applied models featured in the included papers (Fig 5). We aim to provide a comprehensive analysis by considering both their strengths and weaknesses. This balanced examination will help us understand the effectiveness and limitations of each model, thereby guiding the selection of the most appropriate techniques for our research objectives.

$$Predictive\ Models\ of\ Dysgraphia\ Detection \begin{cases} SVM \\ KNN \\ RF \\ K-Means \\ AdaBoost \\ CNN \end{cases}$$

Fig 5 The overview of existing models in Dysgraphia detection

After:

3.5. Comparison of mostly used Predictive Models

This section will discuss the most widely applied predictive models of dysgraphia detection featured in the included papers: SVM, KNN, RF, K-Means, AdaBoost, and CNN. We aim to provide a comprehensive analysis by considering both their strengths and weaknesses. This balanced examination will help us understand each model's effectiveness and limitations, thereby guiding the selection of the most appropriate techniques for our research objectives.

----

5. While the acknowledgment of metric heterogeneity and the table of performance indicators are valuable, this crucial aspect warrants deeper analysis. Consider expanding this section to include: brief explanations of each metric and their relevance to dysgraphia diagnosis; trends in metric usage across studies; implications of this heterogeneity for the field's progress; context-specific considerations for dysgraphia evaluation; and potential future directions for standardization. Additionally, discuss how metric choices relate to clinical relevance and practical application. Enhancing this discussion will provide readers with a more comprehensive understanding of the challenges and opportunities in evaluating AI methods for dysgraphia diagnosis, significantly strengthening your scoping review.

We have expanded the discussion on evaluation metrics by providing detailed explanations of each metric's relevance to dysgraphia diagnosis and their implications for research consistency and comparability. We've also addressed trends in metric usage across studies and discussed the impact of metric heterogeneity on the field's advancement. These additions aim to deepen the understanding of the challenges and opportunities in evaluating AI methods for dysgraphia diagnosis, enriching the manuscript significantly. To address this, we have incorporated the following text into **Section 4.1.2. Extent of Information Uncovered**:

To address this, each metric's relevance to dysgraphia diagnosis is crucial: Accuracy measures the overall correctness of the model, ROC (Receiver Operating Characteristic) curve illustrates the diagnostic ability of a binary classifier system as its discrimination threshold is varied, AUC (Area Under the Curve) represents the degree of separability achieved by the model, and MCC (Matthews Correlation Coefficient) provides a balanced measure of the quality of binary classifications, robust to class imbalance. These metrics are essential for assessing different aspects of model

performance, tailoring their use according to specific research questions or clinical needs in dysgraphia diagnosis. Trends in metric usage across studies indicate a preference for accuracy and AUC in studies focusing on large datasets, where the goal is to maximize both the sensitivity and specificity of detection algorithms. However, MCC is increasingly favored in studies with imbalanced datasets, highlighting its importance in contexts where the negative class (non-dysgraphic individuals) significantly outnumbers the positive class (dysgraphic individuals). The implications of this metric heterogeneity extend beyond mere academic concern; they critically impact the field's progress. Diverse metric use complicates synthesizing findings across studies, potentially slowing the advancement of standardized diagnostic models in dysgraphia. Moreover, context-specific considerations like the subjectivity of handwriting assessments and variability in handwriting across demographic groups underscore the necessity for adopting metrics that can accommodate such diversities.

Additionally, in response to potential concerns about the comprehensiveness of our literature review, we have acknowledged the limitations of our database search range. We have discussed the implications of potentially missing relevant studies from databases not included or unpublished works. To address this, we have added the following sentence to the end of **Section 4.1.2. Extent of Information Uncovered**:

Furthermore, the range of databases searched was limited. While major databases like PubMed, Scopus, Web of Science, IEEE Xplore, and SpringerLink were included, the potential omission of relevant studies from other databases or unpublished works is a critical gap that broader, systematic searches in future studies could mitigate.

Future studies may benefit from broader, systematic searches to ensure a more comprehensive collection of data, which could help mitigate this gap and enhance the robustness of the review.

----

6. The review provides a comprehensive overview of AI algorithms used in dysgraphia detection. However, a critical component is missing: an in-depth analysis of the features used in these models. Given that many methods employ traditional machine learning approaches, as mentioned in your review, a thorough examination of input features is essential. This analysis would significantly enhance the review's value and align with your stated goals of identifying effective AI-based models and understanding associated challenges. Add a dedicated section on feature analysis. Categorize and discuss types of features used (e.g., temporal, spatial, pressure-based, kinematic). Analyse trends in feature selection across studies. Examine the relationship between feature choices and model performance.

We have incorporated your feedback by adding a new section (3.6. Feature Analysis) that provides an in-depth examination of the features used in AI-based models for dysgraphia diagnosis. This section categorizes features into temporal, spatial, pressure-based, and kinematic, analyzing their impact on model performance and selection trends across studies. By highlighting the relationship between feature choices and model efficacy, this addition strengthens the discussion on the role of feature selection in AI-driven dysgraphia assessment.

Below is the newly added section:

3.6. Feature Analysis

Feature selection plays a pivotal role in the effectiveness of AI-based models for dysgraphia diagnosis. The quality and type of features extracted from handwriting samples significantly influence the accuracy and efficiency of these models. This section analyzes the various features utilized in the reviewed studies, categorizing them based on their types and discussing their implications on model performance.

3.6.1. Categorization of Features

AI models for dysgraphia leverage a variety of features derived from handwriting samples. These features can be broadly classified into four categories:
3.6.1.1. Temporal Features: These features relate to the timing aspects of handwriting, such as the duration of writing strokes and the time intervals between strokes. Temporal data is crucial for models that analyze the rhythm and pace of handwriting, which can indicate dysgraphia.
3.6.1.2. Spatial Features: Spatial features include the geometric characteristics of handwriting, such as letter size, shape, and spacing between characters and words. These features help identify irregular handwriting patterns that are common among individuals with dysgraphia.
3.6.1.3 Pressure-Based Features: Pressure features measure the force exerted at different points of the writing instrument during handwriting. Variations in pressure and their patterns can provide insights into fine motor skills, often compromised in individuals with dysgraphia.
3.6.1.4 Kinematic Features: Kinematic features capture the motion involved in handwriting, including the speed and acceleration of writing movements. These features are essential for models that assess the fluidity and smoothness of handwriting motions.

3.6.2. Trends in Feature Selection

The selection of features in AI models often depends on the study's specific objectives and the nature of the data available. Trends observed across the reviewed studies include:

a) **Temporal and kinematic features** are heavily utilized in models focusing on handwriting dynamics. These models are particularly effective in cases where dysgraphia affects motor control during writing.
b) **Spatial and pressure-based features** are common in studies aiming to diagnose dysgraphia through static analysis of written samples, where the shape and pressure patterns provide significant diagnostic clues.

3.6.3. Relationship Between Features and Model Performance

The performance of AI models in diagnosing dysgraphia is highly dependent on the appropriateness and quality of the features used. For instance:

a) Models using a combination of spatial and pressure-based features tend to be more accurate in diagnosing dysgraphia, as these features comprehensively capture both the visual and tactile aspects of handwriting.
b) The inclusion of temporal and kinematic data has been shown to enhance the sensitivity of models, which is particularly useful in early detection scenarios where subtle motor difficulties are present.

The thoughtful selection and combination of features are critical for developing robust AI-based models for dysgraphia diagnosis. Future research should focus on exploring new features and improving feature extraction techniques to further enhance these models' diagnostic capabilities. Moreover, standardizing the features used across studies could lead to more consistent and reliable models, facilitating better comparisons and more definitive conclusions about the effectiveness of different AI approaches.

----

7. While the current review provides valuable insights into AI-based models for dysgraphia diagnosis, the Discussion and Limitations sections could benefit from deeper analysis and more comprehensive treatment. These sections are crucial for contextualizing findings, critically evaluating the current state of research, and guiding future work. Consider expanding these sections to include: a more nuanced interpretation of the results in light of current clinical practices; a critical comparison of different AI approaches and their relative strengths and weaknesses; a thorough examination of methodological limitations across studies; discussion of potential biases in current research; and analysis of gaps between laboratory findings and real-world application.

We have enriched the **Discussion** section by incorporating a nuanced interpretation of AI model results in the context of current clinical practices. This includes a comparative analysis of CNNs and SVMs, highlighting their respective strengths and weaknesses, and how they integrate into

existing diagnostic frameworks. Additionally, we critically examined the methodological limitations and potential biases within the studies reviewed. This expanded discussion aims to provide a deeper understanding of the implications and practical applications of AI models in dysgraphia diagnosis, bridging the gap between theoretical research and clinical utility. To this end, we have added the following text to Section 4. Discussion):

A more nuanced interpretation of these results shows that while AI models such as CNNs and SVMs offer substantial accuracy, their effectiveness should be contextualized within current clinical practices. Clinicians must consider how these AI tools could mesh with existing diagnostic frameworks to enhance or streamline dysgraphia identification without supplanting necessary human judgment. This consideration is crucial for ensuring that AI complements rather than replaces traditional assessment methods, maintaining a balance between technological advancement and clinical intuition. Critically comparing different AI approaches, CNNs often excel in image-based analysis due to their ability to learn complex patterns from data, making them ideal for analyzing handwriting intricacies. However, SVMs might be preferred in scenarios with fewer data samples, or more straightforward decision boundaries are sufficient, highlighting a trade-off between model complexity and interpretability.
Across the studies, methodological limitations such as sample size, diversity, and validation methods have varied, impacting the generalizability of results. These variations suggest a need for standardized research protocols to better compare outcomes across studies. Moreover, potential biases in AI research, such as those introduced by the selection of datasets or the choice of performance metrics, can affect the perceived efficacy of these models. A critical examination of these biases is essential for advancing the field in a meaningful way. Finally, the gap between laboratory findings and real-world applications remains a significant challenge. The controlled environments in which many AI models are developed and tested do not always accurately reflect the complex realities of educational and clinical settings. Bridging this gap requires ongoing collaboration between developers, researchers, and practitioners to ensure these tools are adaptable and practical in everyday use.

Additionally, we have extensively revised the **Limitations** section to provide a more detailed discussion of the methodological challenges and research biases that impact the generalizability and applicability of findings. This revision addresses issues such as missing or unclear dataset information, the subjective nature of certain assessments, and the limited range of databases searched. Furthermore, we expanded our analysis to critically compare different AI approaches, discuss potential biases in current research, and highlight gaps between laboratory findings and their real-world application. These enhancements provide a deeper understanding of the underlying issues and offer a clearer path for future research directions. For this purpose, we added a new section, **4.1.3. Methodological and Research Biases**:

4.1.3. Methodological and Research Biases

A more nuanced interpretation of the results in light of current clinical practices reveals that while AI models are highly effective under controlled study conditions, their application in typical clinical settings may not always align with traditional diagnostic methods. This misalignment necessitates a careful integration strategy to ensure that AI tools augment rather than replace the clinician's expertise. A critical comparison of different AI approaches, such as deep learning models like CNNs versus more traditional machine learning models like SVMs, highlights that while CNNs provide higher accuracy in image-based analyses due to their complex pattern recognition capabilities, SVMs offer advantages in terms of computational efficiency and transparency in decision-making. Examining methodological limitations across studies highlights issues such as the small and non-diverse sample sizes, which limit the extrapolation of findings to a broader population. Additionally, many studies do not undergo external validation, raising concerns about the robustness and reproducibility of the findings.
Discussion of potential biases in current research points to a prevalence of publication bias, where studies with positive findings are more likely to be published, and selection bias, where the data used may not adequately represent the target population. An analysis of gaps between laboratory findings and real-world application indicates that AI models often perform exceptionally well in controlled environments but may fail to deliver similar results in real-world settings. This discrepancy can be attributed to the complex and variable nature of real-world environments compared to the controlled conditions of most studies.

Minor comments : Improve the presentation language and make sure the manuscript is free from typos

We have carefully reviewed the manuscript to improve the presentation language and ensure that it is free from typos. All necessary revisions have been made, and the updated manuscript reflects these changes. These revisions can be clearly seen in the 'Revised Manuscript with Track Changes' file, where all corrections have been indicated.
