# Supplementary material for: AI-driven approaches for dysgraphia diagnosis using online and offline handwriting data: A comprehensive scoping review
Source: PLoS One. 2025 Dec 31;20(12):e0328722. doi: 10.1371/journal.pone.0328722 (PMC12755808; doi:10.1371/journal.pone.0328722)
Supplement: Attachment — Submitted filename: Response to Reviewers.pdf [file pone.0328722.s006.pdf]

Note: To enhance clarity, we have highlighted the reviewers' comments and used **Arial font** for them, while our responses are provided in **Times New Roman**.

Third Revision

Reviewer #2: I appreciate your thorough revisions and the improvements made throughout the manuscript. Most of my earlier comments have been addressed satisfactorily.

However, one key issue remains regarding the treatment of related literature in Section 3.2. In your response, you mention prior reviews in a general sense, but you do not cite or engage directly with the recent and highly relevant review by Kunhoth et al. (2024), "Automated systems for diagnosis of dysgraphia in children: a survey and novel framework". This work overlaps closely in scope with your own and offers a comprehensive review of AI-based dysgraphia diagnosis, including both traditional and deep learning approaches, feature-level discussions, and even non-ML tools.

I strongly encourage you to cite this work explicitly and include a short structured comparison that clarifies how your review builds upon or differs from it. This will improve the scholarly positioning and rigor of your manuscript.

Once this addition is made, I believe the manuscript will be in strong shape for publication.

Our response:

Thank you for highlighting the importance of comparing our work with other comprehensive reviews. In response, we have expanded the discussion in the related work section to include a detailed comparison with the recent review by **Kunhoth et al., 2024** [1], which offers valuable insights into learning disorders and predictive methods, both machine learning and non-machine learning based.

We clarify that while Kunhoth et al. provide a broad overview and effective categorization across various disorders, our review focuses specifically on dysgraphia and delivers more in-depth technical analysis, including a detailed breakdown of individual models, feature selection, and implementation strategies. We emphasize how our review contributes beyond general knowledge by offering model-level insights, highlighting recent AI advancements, and synthesizing results from studies involving modern, hybrid approaches like the integration of spatial and dynamic handwriting features.

Before:

“The publication dates span nearly a decade, from 2015 to the present (Fig 3). Of the 21 selected papers, 8 (n = 8, 38%) [34,35,38,42-44,47] are conference papers, and the remaining 13 (n = 13, 62%) [20,31-33,36,39,40,41,45,46,48-57] are journal articles. The papers originate from various countries, including India, Qatar, Spain, Switzerland, Slovakia, the Czech Republic, Israel, France, the USA, Malaysia, Morocco, and Austria. Additionally, some papers are collaborative efforts involving researchers from multiple nationalities. In addition, only nine of the included studies are open-access (n = 9, 42%) [20,31-33,40,41,45,49], and the 12 other papers are private (n = 12, 58%) [34-39,42-44,46-48].

While our review provides detailed insights into the current AI methodologies for diagnosing dysgraphia, it is essential to contextualize this within the broader academic dialogue. Previous reviews have provided foundational knowledge in this field; however, most adopt a clinical perspective, prioritizing diagnostic criteria and behavioral assessments over AI's potential to transform dysgraphia diagnosis [50,51]. Moreover, few are dedicated solely to dysgraphia, often embedding it within broader discussions of specific learning disabilities (SLDs) like dyslexia and dyscalculia, thus diluting focus on dysgraphia-specific challenges [52-54]. Even reviews exploring AI applications, such as [55,56], typically emphasize traditional machine learning methods (e.g., SVM, AdaBoost) and overlook state-of-the-art approaches, notably the integration of traditional and modern techniques, such as combining handwriting image analysis with dynamic features like pen pressure or motor patterns, which our review examines in detail. Additionally, many prior reviews are outdated, failing to reflect recent AI advancements. Our work addresses these gaps by providing a dysgraphia-specific, up-to-date synthesis of AI applications, comprehensively covering both traditional and cutting-edge methods. This contribution highlights the multidisciplinary relevance of AI in dysgraphia diagnosis through handwritten image analysis, underscoring its potential impact across educational and healthcare settings. Despite growing interest, a recent slowdown in publications suggests a bottleneck in data availability, emphasizing the need for improved data collection and sharing to sustain research momentum in this critical area."

After:

"The publication dates span nearly a decade, from 2015 to the present (Fig 3). Of the 21 selected papers, 8 (n = 8, 38%) [34,35,38,42-44,47] are conference papers, and the remaining 13 (n = 13, 62%) [20,31-33,36,39,40,41,45,46,48-58] are journal articles. The papers originate from various countries, including India, Qatar, Spain, Switzerland, Slovakia, the Czech Republic, Israel, France, the USA, Malaysia, Morocco, and Austria. Additionally, some papers are collaborative efforts involving researchers from multiple nationalities. In addition, only nine of the included studies are open-access (n = 9, 42%) [20,31-33,40,41,45,49], and the 12 other papers are private (n = 12, 58%) [34-39,42-44,46-48].

While our review provides detailed insights into the current AI methodologies for diagnosing dysgraphia, it is essential to contextualize this within the broader academic dialogue. Previous reviews have provided foundational knowledge in this field; however, most adopt a clinical perspective, prioritizing diagnostic criteria and behavioral assessments over AI's potential to transform dysgraphia diagnosis [50,51]. Moreover, few are dedicated solely to dysgraphia, often embedding it within broader discussions of specific learning disabilities (SLDs) like dyslexia and dyscalculia, thus diluting focus on dysgraphia-specific challenges [52-54]. Even reviews exploring AI applications, such as [55,56], typically emphasize traditional machine learning methods (e.g., SVM, AdaBoost) and overlook state-of-the-art approaches, notably the integration of traditional and modern techniques, such as combining handwriting image analysis with dynamic features like pen pressure or motor patterns, which our review examines in detail. Additionally, many prior reviews are outdated, failing to reflect recent AI advancements. One of the more comprehensive recent works is the review by Kunhoth et al. (2024) [57], which provides a broad overview of various learning disorders and explores both machine learning and non-machine learning approaches for their prediction. While their work offers valuable general insights and effectively groups and compares different models, it lacks the depth of analysis found in our study. In contrast, our review includes more specific technical details, considers a wider array of models, and delves into how they are implemented. It also provides deeper guidance and recommendations, making it more actionable for researchers and practitioners. Together, these additions make our review not only complementary to previous works but also a necessary update for the current state of AI research in this domain. Our work addresses these gaps by providing a dysgraphia-specific, up-to-date synthesis of AI applications, comprehensively covering both traditional and cutting-edge methods. This contribution highlights the multidisciplinary relevance of AI in dysgraphia diagnosis through handwritten image analysis, underscoring its potential impact across educational and healthcare settings. Despite growing interest, a recent slowdown in publications suggests a bottleneck in data availability, emphasizing the need for improved data collection and sharing to sustain research momentum in this critical area."

Other Minor Changes:

As part of the future recommendations, we have added an example [2] from our recently published work to demonstrate the practical implementation of integrating fusion models with traditional approaches. This real-world case enhances the clarity and applicability of the proposed direction, offering readers a concrete understanding of how such integration can be effectively realized.

References:

1. Kunhoth, J., Al-Maadeed, S., Kunhoth, S., Akbari, Y., & Saleh, M. (2024). Automated systems for diagnosis of dysgraphia in children: a survey and novel framework. *International Journal on Document Analysis and Recognition (IJDAR)*, *27*(4), 707-735.
2. Vakili, Y. Z., Fallah, A., Esmaeili, K., & Mirfazeli, F. S. (2025). Revolutionizing Dysgraphia Detection: Combining Feature Fusion with Non-Discriminatory Regularization. 2025 11th International Conference on Web Research (ICWR), 250–256. https://doi.org/10.1109/ICWR65219.2025.11006233
